# SERINC5 Inhibits the Secretion of Complete and Genome-Free Hepatitis B Virions Through Interfering With the Glycosylation of the HBV Envelope

**DOI:** 10.3389/fmicb.2020.00697

**Published:** 2020-04-30

**Authors:** Yue Liu, Hong Wang, Jun Zhang, Jing Yang, Lu Bai, Baisong Zheng, Tianhang Zheng, Yingchao Wang, Jianhua Li, Wenyan Zhang

**Affiliations:** ^1^Institute of Virology and AIDS Research, The First Hospital of Jilin University, Changchun, China; ^2^Department of Echocardiography, The First Hospital of Jilin University, Changchun, China; ^3^Key Laboratory of Medical Molecular Virology, Ministry of Education and Health, School of Basic Medical Sciences, Shanghai Medical College of Fudan University, Shanghai, China; ^4^Department of Hepatobiliary Pancreatic Surgery, The First Hospital of Jilin University, Changchun, China

**Keywords:** hepatitis B virus (HBV), serine incorporator 5 (SERINC5), secretion, inhibitory mechanism, glycosylation, host–pathogen interaction

## Abstract

Serine incorporator 3 (SERINC3) and SERINC5 were recently identified as host intrinsic factors against human immunodeficiency virus (HIV)-1 and counteracted by HIV-1 Nef. However, whether they inhibit hepatitis B virus (HBV), which is a severe health problem worldwide, is unknown. Here, we demonstrate that SERINC5 potently inhibited HBV virion secretion in the supernatant without affecting intracellular core particle-associated DNA and the total RNA, but SERINC3 and SERINC1 did not. Further investigation discovered that SERINC5 increased the non-glycosylation of LHB, MHB, and SHB proteins of HBV and slightly decreased HBs proteins levels, which led to the decreased HBV secretion. Importantly, SERINC5 co-localized with LHB proteins in the Golgi apparatus, which is important for glycan processing and transport. In addition, we determined the functional domain in SERINC5 required for HBV inhibition, which was completely different from that required for HIV-1 restriction, whereas phosphorylation and glycosylation sites in SERINC5 were dispensable for HBV restriction. Taken together, our results demonstrate that SERINC5 suppresses HBV virion secretion through interfering with the glycosylation of HBV proteins, suggesting that SERINC5 might possess broad-spectrum antiviral activity.

## Introduction

Hepatitis B virus (HBV) is a hepatotropic virus and the smallest partially double-stranded DNA virus that replicates *via* reverse transcription. HBV infection remains a major public health problem, with more than 240 million chronically infected people worldwide (Schweitzer et al., 2015), which causes severe liver disease, including liver cirrhosis and hepatocellular cancer. Upon HBV infection, relaxed circular DNA (rcDNA) is delivered into the nucleus, where it forms covalently closed circular DNA (cccDNA), which serves as a template for the transcription of pre-genomic RNA (pgRNA) and other viral genes (Levrero et al., 2009). HBV is an enveloped virus that expresses three co-terminal proteins, large (L), middle (M), and small (S), which are important in the viral life cycle. All three envelope proteins contain a common N-linked glycosylation site at N146 in the S domain, while M possesses an additional site at N4 of the pre-S2 domain. Accumulating evidences showed that N-linked glycosylation and the first step in glycan processing pathway are necessary for virion but not subviral particle secretion (Block et al., 1994; Lu et al., 1995; Mehta et al., 1997; Julithe et al., 2014; Bi and Tong, 2018). The L and S proteins are essential for virion formation, while the role of M is still a subject of debate; its presence might enhance the efficiency of virion secretion (Bruss and Ganem, 1991; Ueda et al., 1991). Werr and Prange (1998) demonstrated that a single specific N4 glycan for M is required for M envelope subviral particle secretion, while N146 glycan common to all three envelope proteins is not involved in subviral particle release. Following studies showed that impaired virion secretion by HBV immune escape mutants can be rescued by an extra glycosylation site (Ito et al., 2010; Kwei et al., 2013). The envelope proteins were inserted into the endoplasmic reticulum (ER) membrane and processed at pre-Golgi membranes, where cytosolic capsids are packaged by envelope proteins, that trigger virion assembly and budding reaction (Huovila et al., 1992; Werr and Prange, 1998).

Over the past decade, host restriction factors such as APOBEC3, SAMHD1, tetherin/BST-2, and Mx2 were discovered as host cell barriers against human immunodeficiency virus (HIV) replication, and viruses have developed various strategies to antagonize these restriction factors (Goujon et al., 2013; Kane et al., 2013; Simon et al., 2015; Ghimire et al., 2018). In 2015, two elegant studies discovered that the host cell proteins serine incorporator 3 (SERINC3) and SERINC5 impair HIV-1 infectivity, and HIV-1 Nef and the glycogag protein of murine leukemia virus (MLV) antagonize their restriction by downregulating SERINC expression on the cell surface and preventing their incorporation into virions (Fackler, 2015; Rosa et al., 2015; Usami et al., 2015; Kmiec et al., 2018; Shi et al., 2018; Wu et al., 2019). However, in some strains, neither Env nor Nef prevents high levels of ectopic SERINC5 from incorporating into HIV-1 particles, and Env but not Nef is able to resist the inhibition of virion-associated SERINC5 (Zhang X. et al., 2017). The studies that followed examined the molecular evolutionary arms race between SERINC and viruses, as well as their mechanism of inhibition, which involves the inhibition of HIV-1 fusion pore formation by selectively inactivating sensitive Env glycoproteins or inducing conformational changes to the HIV-1 Env protein (Chande et al., 2016; Heigele et al., 2016; Murrell et al., 2016; Gonzalez-Enriquez et al., 2017; Sood et al., 2017; Trautz et al., 2017; Schulte et al., 2018). SERINC3 and SERINC5 belong to a family of proteins that facilitate lipid biosynthesis or transport in mammalian cells. The family contains five members, SERINC1 to SERINC5, which share more than 17% amino acid identity (Inuzuka et al., 2005; Igarashi and Kashiwagi, 2010). Because of the limited knowledge of SERINC function, further investigation is clearly needed to discover the functionality mechanism of SERINC proteins (Lubke et al., 2014; Matheson et al., 2015).

Here, we determined that SERINC5, but not SERINC3 and SERINC1, led to the significant downregulation of the hepatitis B surface antigen (HBsAg) level and decreased virion-associated DNA in the supernatant through generating non-glycosylated forms of LHB, MHB, and SHB proteins but without affecting intracellular core particle-associated DNA and the total RNA. Our findings shed light into the inhibition of HBV replication by SERINC5 through a novel mechanism that is not employed to inhibit HIV-1.

## Materials and Methods

### Ethics Statement

To obtain human peripheral blood mononuclear cells (PBMCs), healthy volunteers were recruited. PBMCs were obtained from human blood using Lymphocyte Separating Solution (#GS3701; Genview, Beijing, China). Informed consent was signed by all research participants, and this study was approved by the Ethics Review Committee of the First Hospital of Jilin University.

### Plasmid Construction

Genomic RNA of human was extracted from PBMCs using Trizol (#15596-062; Invitrogen, Carlsbad, CA, United States) and reverse transcribed using the Transcriptor First Strand cDNA Synthesis Kit (#4896866001; Roche, Basel, Switzerland) according to the manufacturer’s protocol. The resulting cDNA was used for amplification of SERINC5-HA, SERINC3-HA, and SERINC1-HA fragments at the C-terminal with HA tagged. The PCR products were subcloned into *Sal*I/*Bam*HI sites of VR1012. All truncated mutants of SERINC5 were constructed from wild-type (WT) plasmid.

pHBV1.3 (genotype D) and HBV env protein–deficient virus (pHBV1.3-ENV^–^) were described previously (Li et al., 2010, 2011). LHB, MHB, and SHB proteins were amplified from HBV expression vector and subcloned into *Hin*dIII*/Bam*HI sites of pCMV-3xflag with C-terminal flag-tagged. The deglycosylation mutant N146A, N4A, and NN4/146AA were constructed by PCR-based mutagenesis using WT plasmid as template. LHB- enhanced cyan fluorescent protein (ECFP) plasmid was amplified from HBV expression vector and subcloned into *Sa*/I/*Bg*/II sites of pECFP-C1 with N-terminal ECFP-tagged. All constructs were verified by sequencing. The primers used for PCR were synthesized in Biotec (Shanghai, China), and the primer sequences are listed in Supplementary Table S1.

The HIV-1 infectious clone pNL4-3 was obtained from the AIDS Research and Reference Reagents Program, Division of AIDS, National Institute of Allergy and Infectious Diseases (NIAID), National Institutes of Health (NIH). Env was amplified from pNL4-3 expression vector and subcloned into *Xba*I*/Bam*HI sites of VR1012. BST-2 with an internal HA tag was cloned as previously described (Lv et al., 2015).

### Chemical Synthesis of siRNA

For the single siRNA-mediated gene silencing experiments, siRNA targeting SERINC5 and the control siRNA were designed according to a previous study (Usami et al., 2015) and purchased from Ribobio (Guangzhou, China). The sequence targeting SERINC5 is CACCGTCTACATCTACTCCTA.

### Cell Culture and Transfection

HEK293T (ATCC catalog no. CRL-11268), HepG2 (ATCC catalog no. 77400), and HepG2.2.15 gifted from the Academy of Military Medical Sciences (Beijing, China) cells were cultured as monolayers in Dulbecco’s modified Eagle’s medium (DMEM) and minimum essential medium (#SH30022.01; Hyclone, Logan, UT, United States) supplemented with 10% heat-inactivated (56°C, 30 min) fetal calf serum (FCS, #10099141; GIBCO BRL, Grand Island, NY, United States) and maintained at 37°C with 5% CO_2_ in a humidified atmosphere. HepG2-NTCP and HepAD38 cells were kindly provided by Zhenghong Yuan (Fudan University).

DNA and siRNA transfection were performed with Lipofectamine 2000 (#11668-019; Invitrogen). In particular, LipoFiter3.0 (#HB-TRLF; Hanbio, Shanghai, China) was used for transfection of HepG2.2.15 cells.

### Viruses and Infection

HBV productive replication was described as previously (Zhang W. et al., 2017) and only established in HepG2-NTCP cells using the HepAD38-derived HBV genotype D virus. The supernatant from HepAD38 cells was collected every 3 days for 12 days, then was filtered through a 0.45 μm mesh and concentrated through a 20% sucrose gradient by ultra-centrifugation at 32,000 g for 18 h, followed by resuspension in the fresh culture medium. For HBV infection, the HepG2-hNTCP cells were incubated with collected HBV virions at 1,000 copies per cell in the presence of 5% polyethylene glycol (PEG) 8000 and 2% dimethyl sulfoxide (DMSO) for 24 h and then rinsed three times with phosphate buffered saline (PBS). To make sure that the HBV infection is successful, the viral HBs antigens were examined before cell extraction in every experiment.

### Antibodies and Reagents

The following antibodies and reagents were used: anti-HA antibody (mouse monoclonal, #901514; Biolegend, San Diego, CA, United States), anti-HA antibody (rabbit polyclonal, #715500; Invitrogen), anti-flag antibody (mouse monoclonal, #F1804; Sigma, New York, NY, United States), anti-SERINC5 antibody (rabbit polyclonal, #27066-1-AP; Proteintech), anti-HIV env(gp120) antibody (goat polyclonal, #919001; Abcam, Cambridge, United Kingdom), anti-HBs (Ad/Ay) antibody (horse polyclonal, #ab9193; Abcam), anti-β-tubulin antibody (mouse monoclonal, #RM2002; Beijing Ray Antibody Biotech, Beijing, China), alkaline phosphatase (AP)-conjugated goat anti-mouse IgG secondary antibody (#115-055-062; Jackson, West Grove, PA, United States), horseradish peroxidase (HRP)-conjugated rabbit anti-goat IgG secondary antibody (#SA00001-4; Proteintech, Chicago, United States), HRP-conjugated goat anti-rabbit IgG secondary antibody (#NC-AP132P; Millipore), HRP-conjugated goat anti-mouse IgG secondary antibody (#NC-AP124P; Millipore), anti-human GM130/GOLGA2 polyclonal antibody (#AF8199; R&D Systems, Minneapolis, MN, United States), Alexa Fluor 568 IgG antibody and Alexa Fluor 647 IgG antibody (#A-11011, #A21448; Invitrogen), SelectFX^TM^ Alexa Fluor^TM^ 488 Endoplasmic Reticulum Labeling Kit (#S34200; Invitrogen), MG132 (#S2619; Selleck, Shanghai, China), thapsigargin (#T9033; Sigma), DMSO (#D8418; Sigma), and tunicamycin (#T7765; Sigma).

### Western Blotting

Briefly, cells were harvested and lysed in 1 × loading buffer followed by separation on a 12% polyacrylamide gel. Proteins were transferred onto a polyvinylidene fluoride (PVDF) membrane. The membranes were blocked and then incubated with primary antibodies, followed by a corresponding AP-conjugated secondary antibody or HRP-conjugated secondary antibody. Proteins that used AP-conjugated secondary antibody were visualized using the substrates nitroblue tetrazolium (NBT) (#N6876; Sigma) and 5-bromo-4-chloro-3-indolyl phosphate (BCIP; #11585002001; Roche). Proteins that used HRP-conjugated secondary antibody were incubated by hypersensitive ECL chemiluminescence detection kit (#B500022; Proteintech) and visualized using the Azure C500 Infrared Imaging System (Azure Biosystems, Dublin, CA, United States).

### Detection of Hepatitis B Surface Antigen

ELISA kits (#0001; KHB, Shanghai, China) according to the manufacturer’s instructions were used to detect the HBsAg in the supernatant. The results were quantified by a microplate reader (BIO-RAD). Each experiment was performed in triplicate and independently repeated three times.

### Southern Blot/Northern Blot

Intracellular HBV core particle-associated DNA was extracted as described previously (Qin et al., 2009; Belloni et al., 2012), then was analyzed by Southern blot. Briefly, cells were lysed in 1% NP-40, 50 mM Tris/HCl (pH 7.5), and 8% sucrose at 37°C for 15 min and then incubated with DNase I (100 μg ml^–1^; Sigma), RNase A (100 μg ml^–1^; Sigma), and 10 mM MgCl_2_ at 37°C for 1 h to degrade the free nucleic acids. After centrifugation at 12,000 g for 5 min, the supernatants were treated with proteinase K (100 μg ml^–1^; Sigma) in a buffer containing 25 mM ethylenediaminetetraacetic acid (EDTA), 150 mM sodium chloride (NaCl), and 1% sodium dodecyl sulfate (SDS) at 37°C overnight. After phenol/chloroform extraction, viral DNA was recovered by ethanol precipitation, dissolved in distilled deionized water. HBV total RNA was extracted by using TRIzol reagent (Invitrogen) according to the manufacturer’s instructions.

Southern blot or Northern blot was performed according to the previous studies (Li et al., 2010; Zhang W. et al., 2017). Viral DNA was separated on a 1.5% agarose gel in 1 × TAE (trishydroxymethylaminomethane–acetic acid–ethylene diamine tetraacetic acid) buffer. The gel was then subjected to denaturalization in a solution containing 0.5 M sodium hydroxide (NaOH) and 1.5 M NaCl, followed by neutralization in a buffer containing 1.5 M NaCl and 1 M Tris-HCl (pH 7.4). The DNA was blotted onto a positively charged nylon membrane (Roche#INYC00010; Millipore) in 20 × SSC (1 × SSC is 0.15 M NaCl plus 0.015 M sodium citrate) buffer, and then hybridized and detected using a DIG Northern Starter Kit (#12039672910; Roche) according to the manufacturer’s instructions. For Northern blot, RNA was separated on a denaturing formaldehyde–1.5% agarose gel in 1 × MOPS running buffer (#C516042; BBI, Shanghai, China), then transferred onto a positively charged nylon membrane, hybridized and detected as described above.

### Separation of Virions by Immunoprecipitation and Quantification of Virion DNA by qPCR

Detailed experimental procedures of HBV virions isolation was described as previously (Garcia et al., 2009; Tsai et al., 2009; Ito et al., 2010; Lazar et al., 2017; Bi and Tong, 2018). Briefly, virions were immunoprecipitated from 1.2 ml culture supernatant by a 3 μl anti-HBs antibody conjugated to protein G beads (#11243233001; Roche) overnight at 4°C. In control groups, samples were incubated with beads only. For virion-associated HBV DNA detection, the precipitate was digested with RQ1 RNase-Free DNase (#M6101, Promega, Madison, WI, United States) and Plasmid-safe DNase I (#E3101K; Epicentre Inc., San Diego, CA, United States) according to the manufacturer’s protocol. The viral DNA was further extracted using the DNeasy Blood & Tissue Kit (#69504; Qiagen, Hilden, Germany) and quantified by qPCR using the following primers (Forward: 5′-CCGTCTGTGCCTTCTCATCTG-3′; Reverse: 5′-AGTCCAAGAGTYCTCTTATGYAAGACCTT-3′).

All qPCR reactions were performed using the FastStart Universal SYBR green Master (Rox) (#491314001; Roche) according to the manufacturer’s protocol. The qPCR assay was performed on a Roche 480 instrument (Roche). The threshold cycle (Ct) value of each sample was calculated.

### Confocal Microscopy

For confocal microscopy, HepG2 cells (20–50% confluent) seeded onto coverslips in six-well plates were transfected with LHB-ECFP, LHB-flag proteins, or SERINC5 alone, or SERINC5 and LHB-ECFP proteins both. At 48 h post-transfection, the cells were fixed with 5% formaldehyde for 10 min and permeabilized with 0.25% Triton X-100 for 5 min, blocked in 10% serum for 20 min, and incubated with primary antibody for 1 h. They were then stained with secondary antibody for 1 h. After washing, filling sheets with anti-fluorescent quencher were used, and the images were taken under a fluorescence microscope. The images were acquired on a Zeiss LSM710 confocal microscope and adjusted with ZEN software (Zeiss).

### Co-immunoprecipitation

Cells were lysed with co-immunoprecipitation (co-IP) lysis buffer at 4°C. After centrifugation, the supernatant was incubated with anti-flag antibody conjugated to protein G beads at 4°C for 4 h. The reaction mixtures were then washed with cold wash buffer and subsequently analyzed by immunoblotting.

### Statistical Analysis

All the statistical data represent three independent experiments and are presented as means ± standard deviations (SDs). Statistical significance of the difference was calculated using Student’s *t-*test. Significant differences are indicated in figures as follows: ^∗^*P* < 0.05, ^∗∗^*P* < 0.01, and ^∗∗∗^*P* < 0.001. NS stands for no significance.

## Results

### SERINC5 but Not SERINC3 or SERINC1 Potently Inhibits Hepatitis B Virus Virion Secretion

**FIGURE 1:**
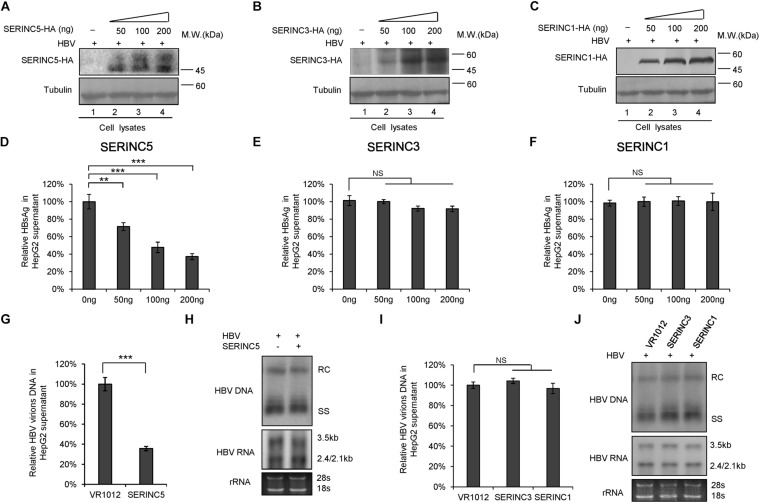
The effect of serine incorporator (SERINC) proteins on hepatitis B virus (HBV) production in HepG2 cells. HepG2 cells were co-transfected with a negative control vector VR1012 or increasing amounts of SERINC5-HA **(A,D)**, SERINC3-HA **(B,E)**, or SERINC1-HA **(C,F)** plus 400 ng pHBV1.3. Cells and culture supernatant were harvested at day 5 post-transfection. **(A–C)** Immunoblot analysis of SERINC proteins using anti-HA antibody, tubulin served as a loading control. **(D–F)** SERINC5, but not SERINC3 and SERINC1, decreased the level of secreted hepatitis B surface antigen (HBsAg) of culture supernatant in a dose-dependent manner by ELISA. HBsAg in the absence of SERINCs were set as 100%. **(G,I)** HBV virion DNA in the supernatant were detected by qPCR following the immunoprecipitation with anti-S Abs conjugated to protein G beads; HBV DNA in the absence of SERINC proteins was set as 100%. **(H,J)** Core particle-associated HBV DNA and the total RNA in cell lysates were detected by Southern blot and Northern blot, respectively. The positions of HBV relaxed circular DNA (RC), single-stranded DNA (SS), and the positions of pregenomic RNA (3.5 kb), pre-S1/S RNA (2.4 kb), and pre-S2/S RNA (2.1) are indicated. 28s and 18s were used as loading controls. **(G–J)** The dose of SERINCs was 200 ng. All of the above results are representative of three independent experiments and presented as the mean ± SD (P-values: ** < 0.01, *** < 0.001). NS, no significance.

To examine the effect of SERINC5, SERINC3, and SERINC1 on the production of HBV particles, HepG2 cells were transfected with increased amounts of SERINC5, SERINC3, or SERINC1 plus an HBV expression vector, as indicated in Figure 1. The expression of SERINC proteins was determined (Figures 1A–C), and increased ectopic SERINC5 inhibited the level of HBsAg in the supernatant in a dose-dependent manner (Figure 1D). In contrast, increased SERINC3 and SERINC1 did not inhibit the level of HBsAg (Figures 1E,F). SERINC5 also inhibited HBV virion-associated DNA in the supernatant which were concentrated by immunoprecipitation with polyclonal anti-HBs antibodies conjugated to protein G, but SERINC3 and SERINC1 did not (Figures 1G,I). HBV DNA from intracellular core particles and the total RNA levels in cell lysates were further detected by Southern blot and Northern blot. The results showed that SERINC5 had no effect on the levels of HBV core particle-associated DNA and the total RNA; SERINC3 and SERINC1 also did not (Figures 1H,J).

**FIGURE 2:**
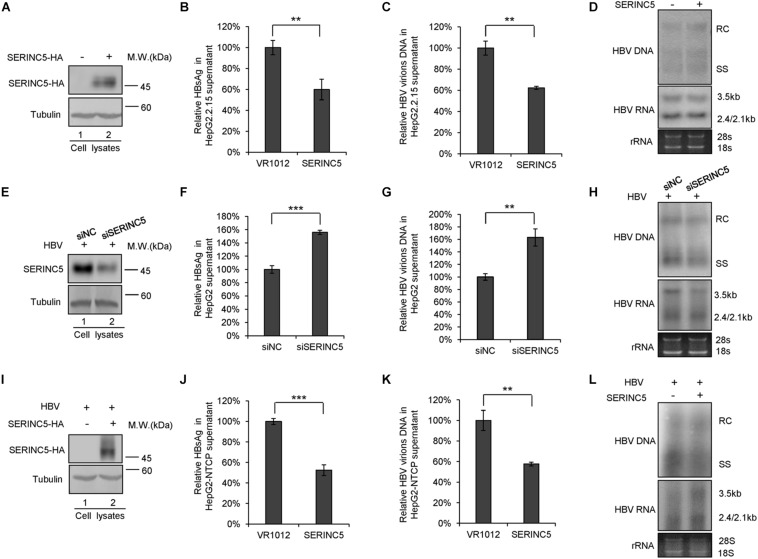
The effect of serine incorporator 5 (SERINC5) on hepatitis B virus (HBV) production in HepG2.2.15 cells. **(A–D)** HepG2.2.15 cells were transfected with a negative control vector VR1012 or SERINC5-HA and harvested for detection at day 3. **(E–H)** The depletion of SERINC5 increased HBV production in HepG2 cells. HepG2 cells were co-transfected with pHBV1.3 and the negative control siRNA (siNC) or siRNA targeting SERINC5 (siSERINC5). **(I–L)** The effect of SERINC5 on HBV production in HepG2-NTCP. HepG2-NTCP cells were transfected with a negative control vector VR1012 or SERINC5-HA at day 1 post-infection with HBV virions from HepAD38. The cells were harvested for detection at day 5. **(A,E,I)** Immunoblot analysis of SERINC5 using anti-HA antibody or anti-SERINC5 antibody, tubulin served as a loading control. **(B,J)** The culture supernatants were monitored by hepatitis B surface antigen (HBsAg) ELISA. HBsAg in the absence of SERINC5 was set as 100%. **(C,K)** Virion DNA from culture supernatant was detected by qPCR following the immunoprecipitation with anti-S Abs conjugated to protein G beads. HBV virions DNA in the absence of SERINC5 were set as 100%. **(D,H,L)** Core particle-associated HBV DNA and the total RNA in cell lysates were detected by Southern blot and Northern blot. All of the above results are representative of three independent experiments and presented as the mean ± SD (*P*-values: ** < 0.01 and *** < 0.001). **(F)** The knockdown of SERINC5 increased the levels of secreted HBsAg in the supernatant. HBsAg in the supernatant treated with siNC was set as 100% (*n* = 3, mean ± SD, ***P < 0.001, paired *t*-test). **(G)** Virion DNA from culture supernatant was detected by qPCR following the immunoprecipitation with anti-S Abs conjugated to protein G beads. HBV DNA in siNC group was set as 100% (*n* = 3, mean ± SD, **P < 0.01, paired *t*-test).

To further confirm the function of SERINC5 in HBV restriction, HepG2.2.15 cells producing HBV virions were employed. SERINC5 was efficiently transfected into HepG2.2.15 cells and well expressed (Figure 2A). The levels of secreted HBsAg and HBV virion-associated DNA in the supernatant were decreased in the presence of SERINC5 by ELISA and RT-qPCR analysis (Figures 2B,C), while the levels of HBV core particle-associated DNA and the total RNA in the cell lysates were not affected by SERINC5 by Southern blot and Northern blot analysis (Figure 2D). Moreover, the knockdown of SERINC5 in HepG2 cells increased the levels of secreted HBsAg and HBV virion-associated DNA in the supernatant compared to that in siNC cells (Figures 2F,G), but not HBV core particle-associated DNA and the total RNA in the cell lysates (Figure 2H). The expression of SERINC5 was confirmed by immunoblot analysis (Figure 2E).

Then, HepG2-NTCP also was employed to detect the effect of SERINC5 on HBV secretion. The HepG2-NTCP cells were infected with HBV virions from HepAD38 cells and transfected with a negative control vector VR1012 or SERINC5 expression vector at day 1 post-infection, and the cells and the supernatant were harvested at day 5 for the detection. The results showed that SERINC5 efficiently inhibited the secretion of HBV virions (Figures 2J,K) but had no effect on the levels of HBV core particle-associated DNA and the total RNA in the cell lysates by Southern blot and Northern blot analysis (Figure 2L). The expression of SERINC5 was confirmed by immunoblot analysis (Figure 2I). Taken together, SERINC5 potentially inhibited the secretion of HBsAg and HBV virions without affecting the intracellular HBV core particle-associated DNA and the total RNA.

### SERINC5 Affects the Expression Pattern of LHB, MHB, and SHB Proteins

**FIGURE 3:**
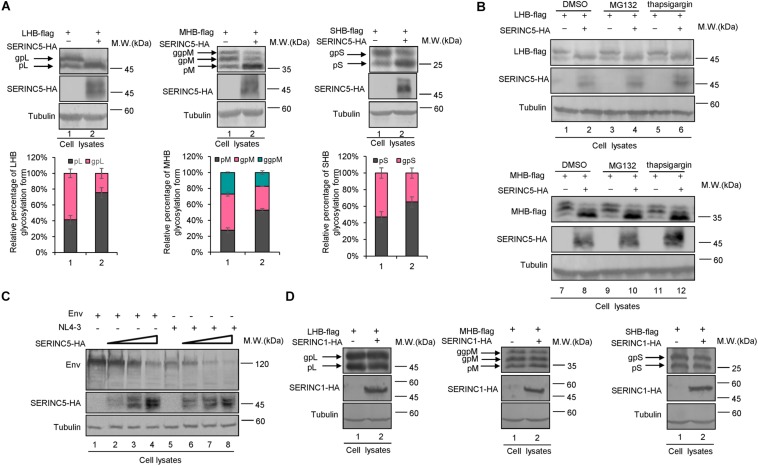
The effect of serine incorporator 5 (SERINC5) on hepatitis B virus (HBV) envelope proteins. **(A)** SERINC5 induced the non-glycosylated LHB, MHB, and SHB proteins in HepG2 cells. HepG2 cells were co-transfected with LHB-flag, MHB-flag, or SHB-flag protein plus a negative control vector VR1012 or the SERINC5-HA. Immunoblot analysis of SERINC5, LHB, MHB, and SHB expression using anti-HA or anti-flag antibodies. The glycosylated (gp or ggp) and non-glycosylated (p) forms of LHB (L), MHB (M), and SHB (S) proteins are indicated. Tubulin served as a loading control. Quantitation of bands corresponding to the proteins of interest was performed using the ImageJ software and were shown below each panel (*n* = 3, mean ± SD). **(B)** Inhibitors MG132 and thapsigargin had no effect on the function of SERINC5-induced the non-glycosylation of LHB and MHB proteins in HepG2 cells. Twelve hours prior to cell harvest, the cells were treated with 10 μM of the proteasome inhibitor MG132 and 3 μM of the autophage-lysosome inhibitor thapsigargin. **(C)** The effect of SERINC5 on HIV-1 Env expression. HEK293T cells were co-transfected with Env or NL4-3 plus a negative control vector VR1012 or SERINC5-HA. Immunoblot analysis of SERINC5 and Env expression using anti-HA or anti-Env antibodies. **(D)** SERINC1 had no effect on the glycosylation of LHB, MHB, and SHB proteins. HepG2 cells were co-transfected with LHB-flag, MHB-flag, or SHB-flag protein plus a negative control vector VR1012 or the SERINC1-HA. Immunoblot analysis of SERINC1, LHB, MHB, and SHB expression using anti-HA or anti-flag antibodies. Tubulin served as a loading control.

Previous studies reported that the inhibition of SERINC5 on HIV-1 induces conformational changes in the HIV-1 Env protein or inactivates sensitive Env glycoproteins (Sood et al., 2017; Schulte et al., 2018). HBs containing LHB, MHB, and SHB are the major components of the HBV envelope, which are required for empty subviral particles and complete virion secretion (Mehta et al., 1997; Garcia et al., 2009; Julithe et al., 2014; Bi and Tong, 2018; Ning et al., 2018). To assess whether SERINC5 affects envelope formation or induces its degradation, HepG2 cells were co-transfected with LHB, MHB, or SHB proteins plus a negative control vector VR1012 or the SERINC5 expression vector (Figure 3A). The expression levels of LHB, MHB, and SHB proteins were slightly decreased, whereas the mobility of LHB, MHB, and SHB was different due to differences in the N-linked glycosylation patterns of LHB, MHB, and SHB proteins in the presence of SERINC5 (Figure 3A). Obviously, the non-glycosylation of LHB, MHB, and SHB was increased in the presence of SERINC5. In addition, the proteasome inhibitor MG132 and the autophage-lysosome inhibitor thapsigargin did not rescue SERINC5-mediated changes in LHB and MHB expression (Figure 3B, lanes 3–6 and 9–12). In HEK293T cells, we also observed a similar phenomenon (Supplementary Figures S1A,B). Interestingly, we observed that SERINC5 induced the decreased HIV-1 env expression (Figure 3C), which is consistent with the observation that some HIV-1 env proteins are sensitive to SERINC5 (Zhang et al., 2019), suggesting that the mechanism of SERINC5 inhibition of HBV is different from that of HIV-1. Further investigation showed that SERINC1 had no effect on the LHB, MHB, and SHB proteins, demonstrating that the effect of SERINC5 on the HBV envelope proteins is specific (Figure 3D). Taken together, SERINC5 might inhibit HBV secretion mainly by affecting the glycosylation of LHB, MHB, and SHB proteins.

### SERINC5 Affects the Glycoslation of LHB, MHB, and SHB Proteins

**FIGURE 4:**
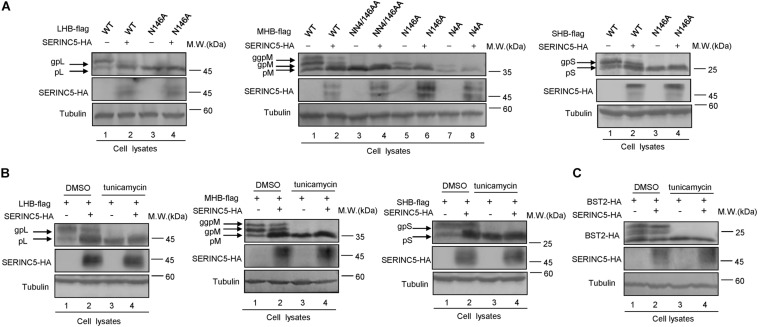
Changes in the serine incorporator 5 (SERINC5)-induced non-glycosylation of hepatitis B virus (HBV) LHB, MHB, and SHB proteins were similar to the pattern of glycan mutants. **(A)** HEK293T cells were co-transfected with LHBs-flag, MHBs-flag, or SHBs-flag proteins or the indicated glycan mutants plus a negative control vector VR1012 or the SERINC5-HA. Immunoblot analysis of SERINC5 and LHB, MHB, SHB, expression using anti-HA or anti-flag antibodies. **(B)** SERINC5 had a similar effect on HBV LHB, MHB, and SHB proteins **(C)** but not BST-2 with tunicamycin. HEK293T cells were co-transfected as indicated. Twelve hours prior to cell harvest, the cells were treated with 10 μg/ml tunicamycin. Immunoblot analysis of SERINC5, LHB, MHB, SHB, and BST-2 expression. Tubulin served as a loading control.

**FIGURE 5:**
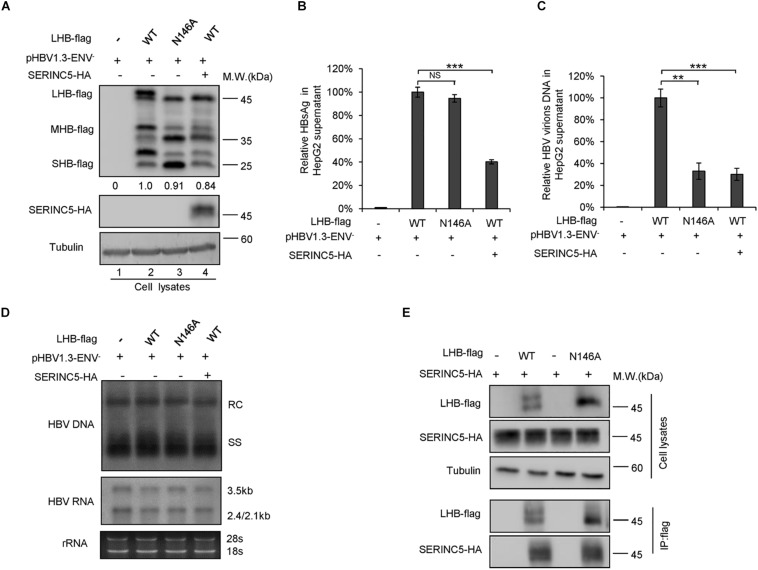
Impacts of serine incorporator 5 (SERINC5)-induced non-glycosylated LHB on virion secretion was similar to those of glycan mutant. HepG2 cells were co-transfected with pHBV1.3-ENV^–^ and LHB-flag proteins or the indicated glycan mutant N146 plus a negative control vector VR1012 or the SERINC5-HA. Cells and culture supernatant were harvested at day 5 post-transfection. **(A)** Immunoblot analysis of SERINC5, LHB wild-type (WT), and N146 mutant expression using anti-HA or anti-flag antibodies; tubulin served as a loading control. Quantitation of bands corresponding to the proteins was analyzed with ImageJ software and was shown below each panel. **(B)** The culture supernatants were monitored by hepatitis B surface antigen (HBsAg) ELISA. HepG2 cells co-transfected with pHBV1.3-ENV^–^ and LHB WT proteins plus a negative control vector VR1012 was set as 100% (*n* = 3, mean ± SD, ***P < 0.001, paired *t*-test). **(C)** Virion DNA from culture supernatant was detected by qPCR following immunoprecipitation with anti-S Abs, HepG2 cells co-transfected with pHBV1.3-ENV^–^ and LHB WT proteins plus a negative control vector VR1012 were set as 100% (*n* = 3, mean ± SD, **P < 0.01, ***P < 0.001, paired *t*-test). **(D)** Quantification of intracellular hepatitis B virus (HBV) DNA and the total RNA in cell lysates were detected by Southern blot and Northern blot. **(E)** LHB and the glycan mutant of LHB proteins interacted with SERINC5 by a co-immunoprecipitation (co-IP) assay. HEK293T cells were co-transfected as indicated. At 48 h post-transfection, the cells were harvested and co-IP with anti-flag plus protein G beads. The cell lysates and co-IP products were analyzed by immunoblotting.

The N-linked glycosylation of HBV envelope proteins plays an important role in viral particle secretion and infectivity. A previous study reported that the imino sugar N-butyldoxynojirimycin (NBDNJ), an inhibitor of the oligosaccharide trimming enzyme α-glucosidase I, and tunicamycin (Heifetz et al., 1979), a global N-glycosylation inhibitor that blocks the transfer of N-acetylglucosamine-1-phosphate to dolichol monophosphate, both suppress the secretion of HBV particles but not subviral particles (Block et al., 1994; Lu et al., 1995). LHB, MHB, and SHB envelope proteins all contain a common N-glycosylation site at 146, while MHB proteins possess an additional N-glycosylation site at N4. The removal of the N146 glycosylation site was detrimental to HBV virion secretion (Julithe et al., 2014). Mehta et al. (1997) demonstrated that N4 glycan site in the pre-S2 domain of MHB proteins also plays a critical role in the secretion of HBV. To further confirm whether SERINC5 generated more non-glycosylated LHB, MHB, and SHB proteins, we constructed a series of mutants bearing single or double glycosylation sites in LHB, MHB, and SHB proteins as indicated. Since the same phenomenon that SERINC5 increased the non-glycosylation of LHB, MHB, and SHB was observed in HEK293T cells (Supplementary Figure S1) as in HepG2 cells, we used HEK293T cells in part of the following experiments. We found that the majority of LHB, MHB, and SHB proteins were non-glycosylated in the presence of SERINC5, which is similar to the pattern of those glycan mutants such as N146A, N4A, or NN4/146AA in LHB, MHB, and SHB proteins (Figure 4A) as well as the effect of tunicamycin on the HBs proteins (Figure 4B). Meanwhile, SERINC5 did not affect BST-2 glycosylation (Figure 4C), suggesting that SERINC5 specifically interferes with the glycosylation of HBV envelope proteins. Moreover, we employed the HBV env protein–deficient virus (pHBV1.3-ENV^–^), which was rendered unable to express all the three envelope proteins (Li et al., 2011), and WT LHB or N146A mutant expression vector to compare the effect of SERINC5 on HBV virion secretion. Due to LHB which entirely contains MHB and SHB, we observed the expression of LHB, MHB, and SHB using LHB expression vector and found that SERINC5 simultaneously induced the non-glycosylation of LHB, MHB, and SHB and slightly decreased the total HBs protein expression levels (84% when compared in the absence of SERINC5) (Figure 5A). As expected, LHB N146A mutant did not decrease the level of secreted HBsAg but decreased the level of HBV virion DNA (Figures 5B,C), consistent with the previous study (Bi and Tong, 2018). However, SERINC5 caused the similar function like LHB N146A mutant in HBV virion DNA level (Figure 5C). Similar with previous data, LHB N146A mutant or SERINC5 all had no effect on the levels of intracellular core particle-associated HBV DNA and the total RNA (Figure 5D).

### Co-localization of SERINC5 With LHB Protein in the Endoplasmic Reticulum and Golgi

**FIGURE 6:**
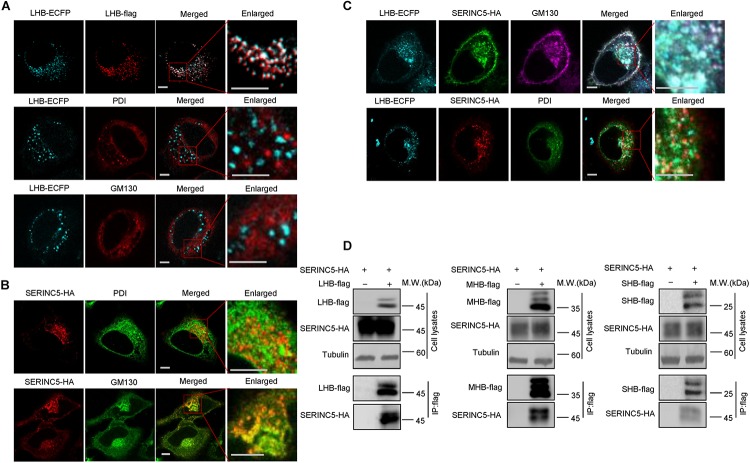
The interaction between serine incorporator 5 (SERINC5) and LHB, MHB, SHB proteins. **(A)** The localization of LHB proteins in HepG2 cells. LHB-ECFP or LHB-flag proteins were transfected into HepG2 cells. Cells were stained with anti-flag, anti-GM130, or anti-PDI antibody 48 h post-transfection. **(B)** The localization of SERINC5 in HepG2 cells. SERINC5-HA was transfected into HepG2 cells. Cells were stained with anti-HA plus anti-GM130 or anti-PDI antibody 48 h post-transfection. **(C)** SERINC5 co-localized with LHB proteins in the Golgi apparatus. HepG2 cells were co-transfected with LHB-ECFP and SERINC5-HA. Cells were stained with anti-HA plus anti-GM130 or anti-PDI antibody 48 h post-transfection. Images were taken with a Zeiss LZM710 confocal microscope. Scale bars: 10 μm. **(D)** LHB, MHB, and SHB proteins interacted with SERINC5 by a co-immunoprecipitation (co-IP) assay. HEK293T cells were co-transfected as indicated. At 48 h post-transfection, the cells were harvested and co-IP with anti-flag plus protein G beads. The cell lysates and co-IP products were analyzed by immunoblotting.

N-linked oligosaccharides are processed within the ER, from which they are transported through the Golgi apparatus required for posttranslational modifications and the trafficking of proteins into extracellular fluid. We next tested whether SERINC5 co-localizes with LHB proteins in the ER or Golgi apparatus using distinct organelle markers, thereby interfering with the glycosylation of HBV envelope proteins. HepG2 cells expressing SERINC5-HA and LHB-ECFP proteins were stained with anti-HA, antibody against GM130, which is a Golgi matrix protein, or antibody against PDI, an ER marker. We first demonstrated that LHB-ECFP and LHB-flag proteins both localized in the cytoplasm, suggesting that LHB-ECFP fusion protein has the same localization with LHB. We also observed that LHB protein alone did not localize within the ER or Golgi apparatus (Figure 6A), while SERINC5 alone was distributed in the Golgi but not the ER (Figure 6B), which is consistent with a previous report^1^. However, LHB proteins were translocated into the Golgi in the presence of SERINC5 and completely co-localized with SERINC5 in the Golgi in the cytoplasm (Figure 6C). A co-immunoprecipitation assay also showed that SERINC5 specifically interacted with LHB, MHB, and SHB proteins (Figure 6D). Interestingly, we observed that SERINC5 also interacted with LHB N146A mutant, further confirming the interaction between SERINC5 and LHB (Figure 5E). These data indicated that SERINC5 interacted with LHB proteins to modify its glycosylation.

### Functional Domains of SERINC5 Are Required for Hepatitis B Virus Restriction

**FIGURE 7:**
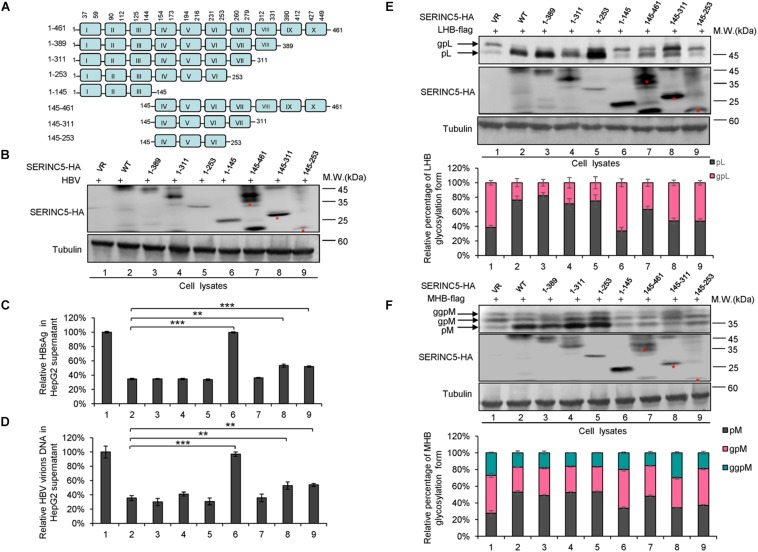
The functional domain of serine incorporator 5 (SERINC5) required for hepatitis B virus (HBV) inhibition. **(A)** Schematic diagram of the transmembrane domains of SERINC5 and its mutants. The structure was predicted by Transmembrane Prediction using Hidden Marow Models (TMHMM) on a public server (http://www.cbs.dtu.dk/services/TMHMM-2.0/). **(B–D)** The effect of wild-type (WT) SERINC5 and SERINC5 truncated mutants on hepatitis B surface antigen (HBsAg) and HBV virion secretion. HepG2 cells were co-transfected as indicated. **(B)** Immunoblot analysis of SERINC5 and SERINC5 mutants using anti-HA antibody, tubulin served as a loading control. **(C)** The culture supernatants were monitored by HBsAg ELISA. HBsAg in the absence of SERINC5 was set as 100%. **(D)** Virion DNA from culture supernatant was detected by qPCR following the immunoprecipitation with anti-S Abs conjugated to protein G beads. HBV virion DNA in the absence of SERINC5 was set as 100% (*n* = 3, mean ± SD, **P < 0.01, ***P < 0.001, paired t-*t*est). The effect of WT SERINC5 and SERINC5 mutants on the glycosylation of LHB **(E)** and MHB **(F)** proteins. HEK293T cells were co-transfected as indicated and then harvested for immunoblot analysis 48 h post-transfection. Immunoblot analysis of SERINC5, LHB, and MHB expression using anti-HA or anti-flag antibodies. The glycosylated (gp or ggp) and non-glycosylated (p) forms of LHB (L), and MHB (M) proteins are indicated. Tubulin served as a loading control. Quantitation of bands corresponding to the proteins of interest was performed using the ImageJ software and was shown below each panel (*n* = 3, mean ± SD).

SERINC5 is a transmembrane protein that contains 10 putative transmembrane helices. To identify the functional domains of SERINC5 important for HBV restriction, we constructed a series of truncated SERINC5 mutants, as indicated in Figure 7A. SERINC5 mutants without 10th domain, even residues 1–253, maintained the ability to inhibit HBV (Figures 7B–D, lanes 3–5), which is inconsistent with data suggesting that the 10th domain is required for the activity of SERINC5 against HIV-1 (Zhang X. et al., 2017). However, SERINC5 mutant comprising residues 1–145 totally and mutants comprising residues 145–311 and 145–253 partially lost the ability to inhibit HBV (Figures 7B–D, lanes 6, 8, 9). The effect of SERINC5 mutants on the glycosylation of LHB and MHB proteins also was examined, and the SERINC5 1–145 mutants completely and 145–311 and 145–253 mutants partially did not produce more non-glycosylated LHB and MHB proteins than WT SERINC5, explaining why the above mutants lost the ability to inhibit HBV secretion (Figures 7E,F). Consistent with a previous study (Zhang X. et al., 2017), we observed that the expression of SERINC5 mutants 1–253 and 145–253 was unstable.

**FIGURE 8:**
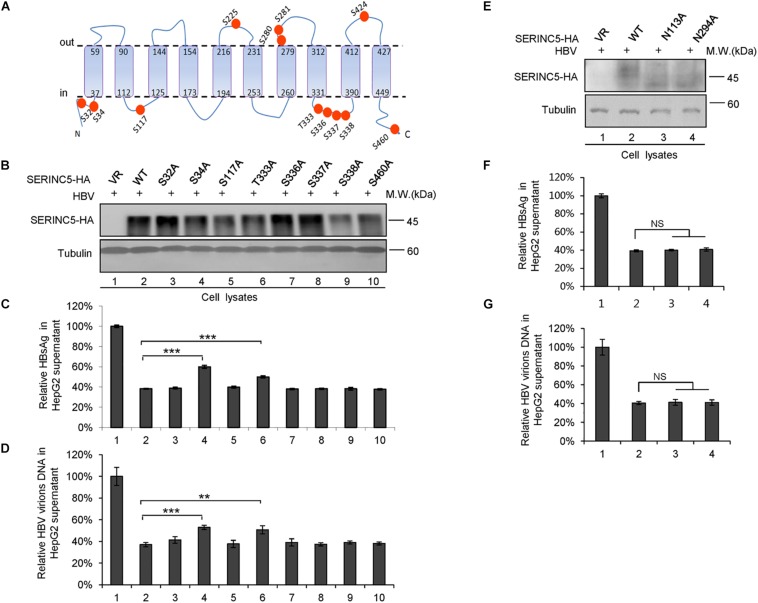
The correlation between phosphorylation and glycosylation of serine incorporator 5 (SERINC5) with hepatitis B virus (HBV) restriction. **(A)** Diagram of the phosphorylation sites in SERINC5. **(B–D)** The effect of wild-type (WT) SERINC5 and SERINC5 phosphorylation mutants on hepatitis B surface antigen (HBsAg) and HBV virion secretion. HepG2 cells were co-transfected as indicated, harvested for immunoblot analysis **(B)**, monitored by HBsAg ELISA **(C)**, and detected by qPCR following the immunoprecipitation **(D)** (*n* = 3, mean ± SD, **P < 0.01, ***P < 0.001, paired *t*-test). Tubulin served as a loading control. **(E–G)** The effect of WT SERINC5 and SERINC5 glycosylation mutants on HBsAg and HBV virion secretion. HepG2 cells were co-transfected as indicated, harvested for immunoblot analysis **(E)**, monitored by HBsAg ELISA **(F)**, and detected by qPCR following the immunoprecipitation **(G)** (*n* = 3, mean ± SD, NS, no significance, paired *t*-test). Tubulin served as a loading control.

We also examined the effect of phosphorylation of SERINC5 on its anti-HBV activity. A series of SERINC5 mutants bearing phosphorylation sites showed no different anti-HBV activity than WT SERINC5, except for the slight effect of the S34A and T333A mutations (Figures 8A–D). By sequence alignment, we further identified two conserved N-glycosylation sites in SERINC5, N133 and N294, and then examined the effect of their mutants on the glycosylation of SERINC5 and HBV restriction. Immunoblot analysis showed that N133 and N294 are the major sites of N-glycosylation in SERINC5 (Figure 8E), but they were not required for HBV restriction (Figures 8F,G). The same phenomena were observed in HEK293T cells (Supplementary Figure S2).

## Discussion

Accumulating evidence has shown that host intrinsic factors, such as APOBEC3G and SAMHD1, possess broad-spectrum antiviral activity against various viruses, including the DNA virus HBV and the RNA virus enterovirus 71, although they were initially shown to act on HIV-1 (Turelli et al., 2004; Chen et al., 2014; Simon et al., 2015; Li et al., 2018). SERINC5 was recently identified as a host antiviral factor; its known antiviral activity has been limited to HIV-1. In this study, we demonstrated that SERINC5 inhibits HBV virion secretion including secreted HBsAg and HBV virion-associated DNA in the supernatant mainly through producing non-glycosylated HBV LHB, MHB, and SHB proteins without affecting intracellular HBV core particle-associated DNA and the total RNA. Although we also observed that SERINCE5 slightly decreased the total expression levels of HBs proteins by ImageJ software (Figure 5A), but that is not a major contributor to decreased HBV secretion. The novel mechanism of SERINC5 against HBV is completely different from its anti-HIV-1 activity, which mainly induces conformational changes in the HIV-1 envelope (Sood et al., 2017; Schulte et al., 2018).

For many viral infections, pathogen glycoproteins are required for entry, successful replication, secretion, and even host immune evasion (Yap et al., 2017; Tzarum et al., 2018). Therefore, glycan processing and some host factors involving glycosylation targeting pathogen proteins modulate virion production. Indeed, the three glycoproteins in HBV, the LHB, MHB, and SHB proteins, are important in HBV life cycle, while the addition of N-glycosylation inhibitors or the N146 glycosylation sites in the LHB, MHB, and SHB proteins impaired HBV virion secretion (Block et al., 1994; Lu et al., 1995; Mehta et al., 1997; Ito et al., 2010; Julithe et al., 2014). Moreover, Ito et al. (2010) found that the I110M, G119E, and R169P mutations in the S domain of viral envelope proteins impair virion secretion through the SHBs. The above studies support our conclusion that SERINC5 restricts HBV through generating non-glycosylated LHB, MHB, and SHB envelope proteins (Figures 3–5), thus affecting the secretion of complete and genome-free virion particles. We also observed the interaction between SERINC5 with LHBs, MHBs, and SHBs, even with LHBs N146A mutant, so the mechanism by which SERINC5 interferes with the glycosylation of HBV envelope proteins might be due to the block of the addition of glycans. The interaction sites of S proteins required for SERINC5 binding are needed to be identified in the future. By co-localization assay, we also observed that LHB proteins originally localized in the cytoplasm were arrested in the Golgi apparatus in the presence of SERINC5, while SERINC5 was mainly distributed in the cell membrane and the Golgi (Figure 6). The result that LHBs alone do not locate in ER or Golgi is consistent with a previous study that the HBsAg lipoprotein particle is assembled from dimeric HBsAg and occurs in an intermediate compartment between the ER and the Golgi (Huovila et al., 1992). However, SERINC5 arrests LHBs in the Golgi through binding to LHB proteins. These results further confirmed the functional mechanism of SERINC5, which induces HBV envelope protein non-glycosylation. Recent studies identified several ER-localized protein complexes essential for Flaviviridae infection by genome-wide screening due to the intense research in this area (Ma et al., 2015; Marceau et al., 2016; Savidis et al., 2016; Puschnik et al., 2017; Hafirassou et al., 2018). In particular, oligosaccharyltransferase (OST) complex subunits were required for flaviviral infection. However, studies on the complex required for the glycosylation of HBV envelope proteins have not been reported until now, and this area needs to be further investigated in the future.

The 10th transmembrane domain in SERINC5 is required for the stable expression of SERINC5 and its restriction of HIV-1, as shown by Zhang X. et al. (2017), while a long cytoplasmic loop connecting helices 7 and 8 governs its sensitivity to HIV-1 Nef (Dai et al., 2018). Here, we found that the 10th transmembrane domain was not necessary for HBV restriction, while truncated mutants comprising residues 1–253 and 145–461 maintained the ability to suppress HBV secretion, indicating that the fourth to sixth domains in SERINC5 are necessary for HBV suppression (Figure 7). The glycosylation sites N113 and N294 in SERINC5 predicted by a web server^2^ in our study were mutated and demonstrated not to be required for its restriction of HBV (Figure 8), which is in line with data indicating that N294 is not required for HIV-1 restriction (Sharma et al., 2018). In this study, we also confirmed the expression of SERINC5 in various cell lines including immortalized kidney cells HEK293T, liver cells HepG2, HBV-producing cells HepG2.2.15, as well as primary liver cells L02 by Western blotting analysis (Supplementary Figure S3), which maintain the similar tendency with mRNA levels as reported (Rosa et al., 2015). As a host anti-HBV restrictive factor, whether the expression level of SERINC5 correlates with HBV viral load or HBV sensitivity *in vitro* is worth to be further investigated in the future.

In summary, this study demonstrated that SERINC5 is a potential anti-HBV intrinsic factor. Therefore, the stimulation or upregulation of SERINC5 may be a novel approach for the development of anti-HBV strategies.

## Data Availability Statement

All datasets generated for this study are included in the article/Supplementary Material.

## Ethics Statement

The studies involving human participants were reviewed and approved by Ethics Review Committee of the First Hospital of Jilin University. The patients/participants provided their written informed consent to participate in this study.

## Author Contributions

WZ and JL conceived and designed the experiments and analyzed the data. WZ, YL, and JZ wrote the manuscript. YL, JZ, JY, LB, and HW performed the experiments. YW, BZ, and TZ contributed reagents, materials, and analysis tools.

## Conflict of Interest

The authors declare that the research was conducted in the absence of any commercial or financial relationships that could be construed as a potential conflict of interest.

## Footnotes

1 https://www.genecards.org

2 http://www.cbs.dtu.dk/services/NetNGlyc/

## Supplementary Material

FIGURE S1 **(A)** SERINC5 induced the non-glycosylated LHB, MHB, and SHB proteins in HEK293T cells. HEK293T cells were co-transfected with LHB-flag, MHB-flag or SHB-flag protein plus a negative control vector VR1012 or the SERINC5-HA. Immunoblot analysis of SERINC5, LHB, MHB, and SHB expression using anti-HA or anti-flag antibodies. The glycosylated (gp or ggp) and non-glycosylated (p) forms of LHB (L), MHB (M), and SHB (S) proteins are indicated. Tubulin was served as a loading control. **(B)** Inhibitors MG132 and thapsigargin had no effect on the function of SERINC5-induced the non-glycosylation of LHB and MHB proteins in HEK293T cells. Twelve hours prior to cell harvest, the cells were treated with 10 μM of the proteasome inhibitor MG132 and 3 μM of the autophage-lysosome inhibitor thapsigargin.

FIGURE S2 The correlation between phosphorylation and glycosylation of SERINC5 with HBV restriction in HEK293T cells. **(A–C)** The effect of WT SERINC5 and SERINC5 phosphorylation mutants on HBsAg and HBV virion secretion. HEK293T cells were co-transfected as indicated, harvested for immunoblot analysis **(A)**, monitored by HBsAg ELISA **(B)** and detected by qPCR following the immunoprecipitation **(C)** (*n* = 3, mean ± SD, ^∗∗^*P* < 0.01, ^∗∗∗^*P* < 0.001, paired *t*-test). Tubulin served as a loading control. **(D–F)** The effect of WT SERINC5 and SERINC5 glycosylation mutants on HBsAg and HBV virion secretion. HEK293T cells were co-transfected as indicated, harvested for immunoblot analysis **(D)**, monitored by HBsAg ELISA **(E)** and detected by qPCR following the immunoprecipitation **(F)**. (*n* = 3, mean ± SD, NS, no significance, paired *t*-test). Tubulin served as a loading control.

FIGURE S3 The protein expression levels of SERINC5 in various cell lines. HepG2, HepG2.2.15, HEK293T, and L02 cells were lysed and loaded for western blotting analysis using SERINC5 antibody. Tubulin was served as a loading control.

TABLE S1 Primers used for plasmid construction in this study.
