# Supplementary figures and images for: SERINC5 Inhibits the Secretion of Complete and Genome-Free Hepatitis B Virions Through Interfering With the Glycosylation of the HBV Envelope
Source: Front Microbiol. 2020 Apr 30;11:697. doi: 10.3389/fmicb.2020.00697 (PMC7216740; doi:10.3389/fmicb.2020.00697)

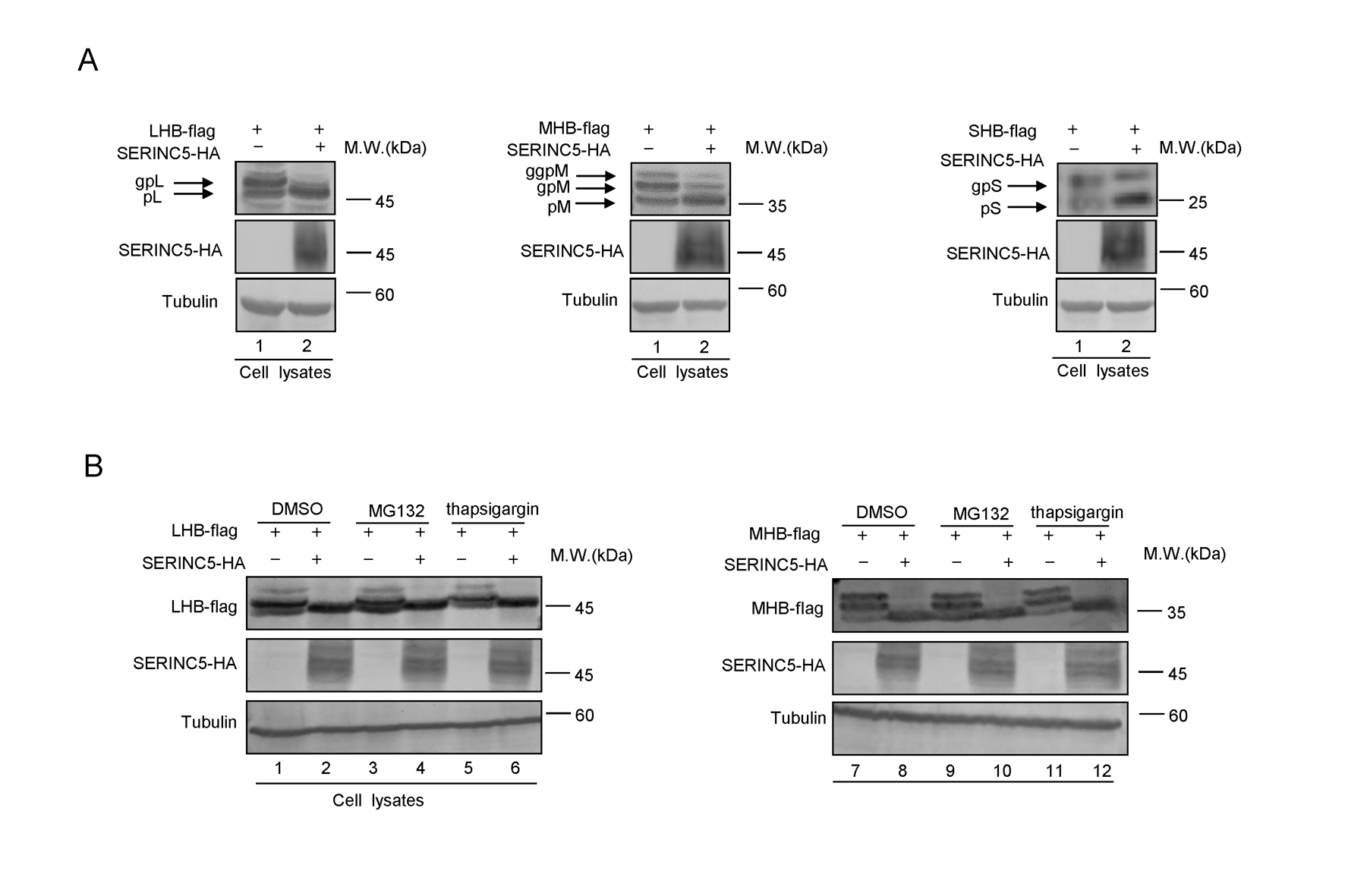

Supplement: FIGURE S1 — (A) SERINC5 induced the non-glycosylated LHB, MHB, and SHB proteins in HEK293T cells. HEK293T cells were co-transfected with LHB-flag, MHB-flag or SHB-flag protein plus a negative control vector VR1012 or the SERINC5-HA. Immunoblot analysis of SERINC5, LHB, MHB, and SHB expression using anti-HA or anti-flag antibodies. The glycosylated (gp or ggp) and non-glycosylated (p) forms of LHB (L), MHB (M), and SHB (S) proteins are indicated. Tubulin was served as a loading control. (B) Inhibitors MG132 and thapsigargin had no effect on the function of SERINC5-induced the non-glycosylation of LHB and MHB proteins in HEK293T cells. Twelve hours prior to cell harvest, the cells were treated with 10 μM of the proteasome inhibitor MG132 and 3 μM of the autophage-lysosome inhibitor thapsigargin. [file Image_1.TIF]

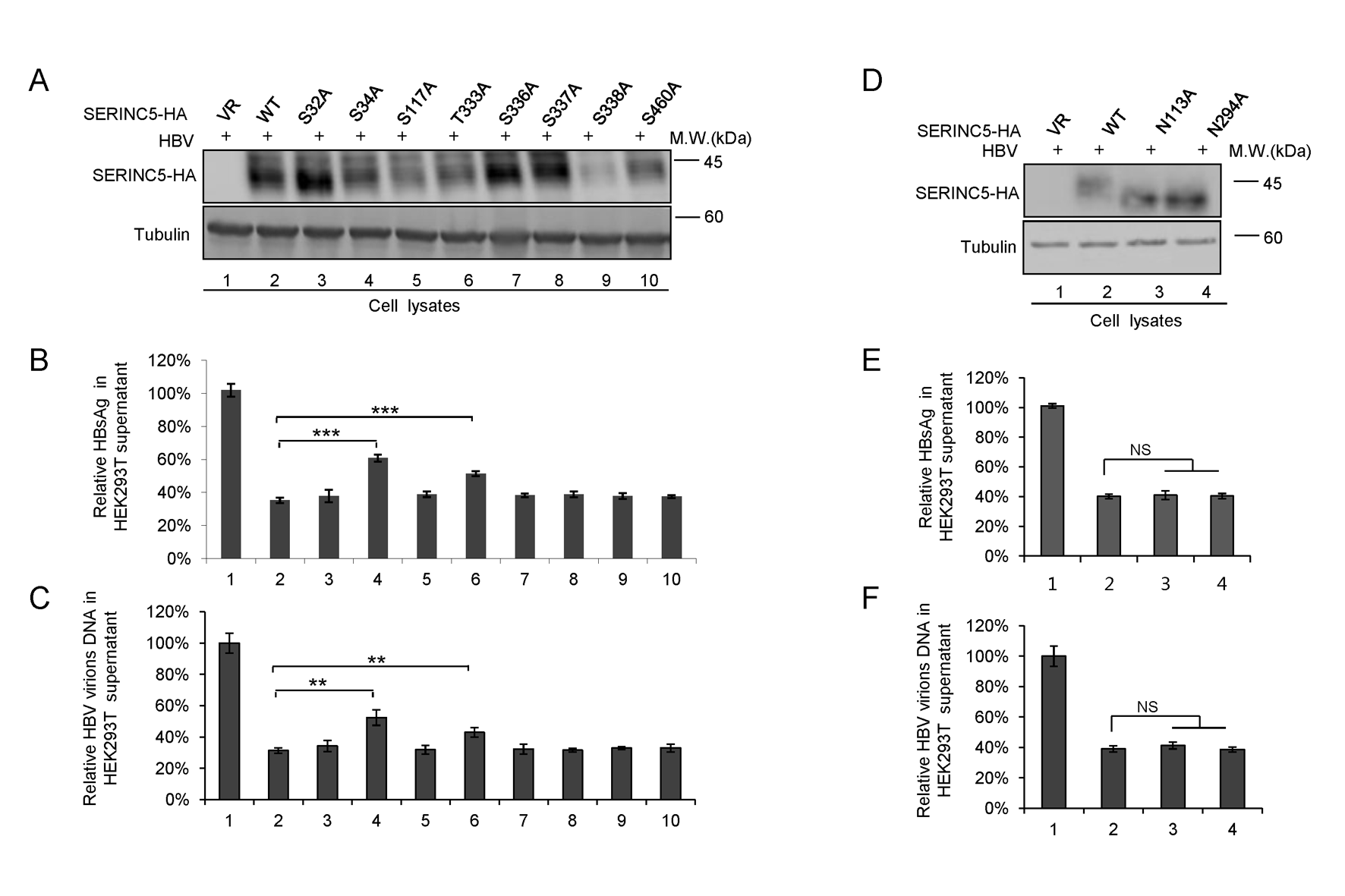

Supplement: FIGURE S2 — The correlation between phosphorylation and glycosylation of SERINC5 with HBV restriction in HEK293T cells. (A–C) The effect of WT SERINC5 and SERINC5 phosphorylation mutants on HBsAg and HBV virion secretion. HEK293T cells were co-transfected as indicated, harvested for immunoblot analysis (A), monitored by HBsAg ELISA (B) and detected by qPCR following the immunoprecipitation (C) (n = 3, mean ± SD, ∗∗P < 0.01, ∗∗∗P < 0.001, paired t-test). Tubulin served as a loading control. (D–F) The effect of WT SERINC5 and SERINC5 glycosylation mutants on HBsAg and HBV virion secretion. HEK293T cells were co-transfected as indicated, harvested for immunoblot analysis (D), monitored by HBsAg ELISA (E) and detected by qPCR following the immunoprecipitation (F). (n = 3, mean ± SD, NS, no significance, paired t-test). Tubulin served as a loading control. [file Image_2.TIF]

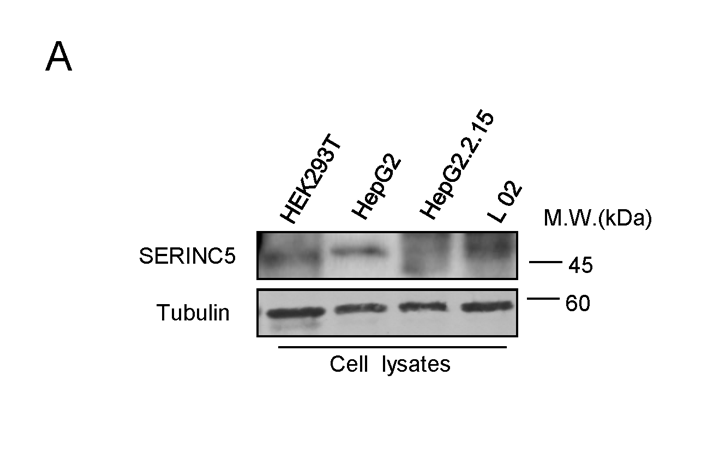

Supplement: FIGURE S3 — The protein expression levels of SERINC5 in various cell lines. HepG2, HepG2.2.15, HEK293T, and L02 cells were lysed and loaded for western blotting analysis using SERINC5 antibody. Tubulin was served as a loading control. [file Image_3.TIF]
