# Supplementary material for: SERINC5 Inhibits the Secretion of Complete and Genome-Free Hepatitis B Virions Through Interfering With the Glycosylation of the HBV Envelope
Source: Front Microbiol. 2020 Apr 30;11:697. doi: 10.3389/fmicb.2020.00697 (PMC7216740; doi:10.3389/fmicb.2020.00697)
Supplement: TABLE S1 — Primers used for plasmid construction in this study. [file Table_1.PDF]

Table S1 Primers used for plasmid construction in this study

Primer name	Primer direction	Sequence (5′–3′)
SERINC1-SalI-F	Forward	GCGTCGACACCATGGGGAGCGTCCTG
SERINC1-HA-BamHI-R	Reverse	CGGGATCCTCACGCGTAATCTGGGACGTCGTAAGGGTAGTCAAAATCACGATTTG
SERINC3-SalI-F	Forward	GCGTCGACACCATGGGGGCTGTGCT
SERINC3-HA-BamHI-R	Reverse	CGGGATCCTCACGCGTAATCTGGGACGTCGTAAGGGTAGCTGAAGTCCCGACTG
SERINC5-SalI-F	Forward	GCGTCGACACCATGTCAGCTCAGTGCTG
SERINC5-HA-BamHI-R	Reverse	CGGGATCCTCACGCGTAATCTGGGACGTCGTAAGGGTACACAGAGAACTCCCGG
SERINC5-HA-145-R	Reverse	CGGGATCCTCACGCGTAATCTGGGACGTCGTAAGGGTATGGAATGAAAGAAAGCTCC
SERINC5-HA-253-R	Reverse	CGGGATCCTCACGCGTAATCTGGGACGTCGTAAGGGTAGATGGCTACCAATGATATA
SERINC5-HA-311-R	Reverse	CGGGATCCTCACGCGTAATCTGGGACGTCGTAAGGGTAGTTTTCATCTCTGTACAGGTC
SERINC5-HA-389-R	Reverse	CGGGATCCTCACGCGTAATCTGGGACGTCGTAAGGGTAGACGGTGCCTTTCTTC
SERINC5-145-F	Forward	GCGTCGACACCATGCCAGATCAGGACACCTTTCTG
SERINC5-S32A-F	Forward	CCCAGGATTCGGCAGGCCCTCAGCAC
SERINC5-S32A-R	Reverse	CCTGCCGAATCCTGGGGCAGCAATCAC
SERINC5-S34A-F	Forward	GATTCGGCAGTCCCTCGCCACCCGCTTC
SERINC5-S34A-R	Reverse	GCGAGGGACTGCCGAATCCTGGGGCAGC
SERINC5-S117A-F	Forward	ATCAACAACAGCAAAGCTTGTAGAGCTC
SERINC5-S117A-R	Reverse	GCTTTGCTGTTGTTGATTTTCAAGGTC
SERINC5-T333A-F	Forward	GTATTCATGTTTGACATCAGCAACAAGATC
SERINC5-T333A-R	Reverse	CTGATGTCAAACATGAATACAAGATACATC
SERINC5-S336A-F	Forward	GACATCAACAACAAGAGCGAGTTCTGAC
SERINC5-S336A-R	Reverse	CTCTTGTTGTTGATGTCAAACATGAATAC
SERINC5-S337A-F	Forward	CATCAACAACAAGAGCGGCTTCTGACGCTC
SERINC5-S337A-R	Reverse	GCCGCTCTTGTTGTTGATGTCAAACATGAATAC
SERINC5-S338A-F	Forward	CAACAACAAGAGCGGCTGCTGACGCTCTG
SERINC5-S338A-R	Reverse	CAGCCGCTCTTGTTGTTGATGTCAAACATG
SERINC5-S460A-F	Forward	CCCCACCCGGGAGTTCGCTGTGTACCCTTACG

SERINC5-S460A-R	Reverse	CGAACTCCCGGGTGGGGCAGCAGAGGG
SERINC5-N113A-F	Forward	CTGACCTTGAAAATCGCCAACAGCAAAAG
SERINC5-N113A-R	Reverse	GCGATTTTCAAGGTCAGTAGACAGAAGATAAAG
SERINC5-N294A-F	Forward	GATGAACATGGGAAAGCTGTTACAATC
SERINC5-N294A-R	Reverse	GCTTTCCCATGTTCATCTAGAACTACTTC
LHBs-HindIII-F	Forward	CCAAGCTTACCATGGGGCAGAATCTTTCC
MHBs-HindIII-F	Forward	CCAAGCTTACCATGCAGTGGAATTCCACAAC
SHBs-HindIII-F	Forward	CCAAGCTTACCATGGAGAACATCACATCAGGA
HBs-BamHI-R	Reverse	CGGGATCCAATGTATACCCAAAGAC
S-N146A-F	Forward	CCAAACCTTCGGACGGAGCTTGCACCTGT
S-N146A-R	Reverse	CGTCCGTCCGAAGGTTTGGTACAGCAACAG
preS2-N4A-F	Forward	GAAGAAGATCTTCAGTGGGCTTCCACAACC
preS2-N4A-R	Reverse	CGCCACTGAAGATCTTCTTCTGATATGAGTTTTT
